# Neuropsychological functioning and quality of life in congenital myopathies: a systematic review of children and caregiver outcomes

**DOI:** 10.3389/fmed.2026.1876262

**Published:** 2026-08-06

**Authors:** Sergio Rinella, Gennaro Anastasio, Annamaria Sapuppo, Manuela Lo Bianco, Roberta Leonardi, Piero Pavone, Martino Ruggieri, Agata Polizzi

**Affiliations:** 1 Unit of Pediatric Clinic, Azienda Ospedaliero-Universitaria Policlinico “G. Rodolico-San Marco”, Department of Clinical and Experimental Medicine, University of Catania, Catania, Italy; 2 Department of Educational Sciences, University of Catania, Catania, Italy; 3 National Center for Rare Diseases, Istituto Superiore di Sanità, Rome, Italy; 4 Unit of Pediatrics and Pediatric Emergency, Azienda Ospedaliero-Universitaria Policlinico “G. Rodolico-San Marco”, San Marco Hospital, University of Catania, Catania, Italy; 5 Postgraduate Training Programme in Pediatrics, Department of Clinical and Experimental Medicine, University of Catania, Catania, Italy

**Keywords:** caregiver burden, cognitive development, congenital myopathies, intellectual functioning, neurodevelopment, neuropsychology, pediatric neuromuscular disorders, quality of life

## Abstract

**Background:**

Congenital myopathies (CMs) are a heterogeneous group of rare or ultra-rare inherited muscular disorders in which cognitive, neuropsychological and psychosocial outcomes remain poorly characterized.

**Objective:**

To synthesize current evidence on cognitive development, neuropsychological and quality-of-life (QoL) outcomes in pediatric CMs, including caregiver burden.

**Methods:**

A systematic review was conducted according to PRISMA guidelines and registered on PROSPERO (CRD420261373780). PubMed, Scopus, Web of Science and ClinicalTrials.gov were searched up to June 2026. Observational studies reporting neuropsychological or psychosocial outcomes in children with CMs were included.

**Results:**

18 studies (104 patients; 56 caregivers) were included, predominantly case reports/series (72.2%). Cognitive outcomes were heterogeneous: most patients showed preserved intellectual functioning; however, beyond the expected motor impairment, some exhibited additional language or domain-specific deficits. More severe profiles were observed in ACTA1-related disease and selected rare genotypes, often associated with brain abnormalities. In contrast, cognition was largely preserved in X-linked myotubular myopathy, although adaptive functioning and QoL were frequently reduced. School outcomes were rarely reported. Caregiver data indicated a substantial and multidimensional burden.

**Conclusion:**

Neuropsychological and psychosocial outcomes in pediatric CMs are clinically relevant but understudied and heterogeneous across genotypes. Standardized, motor-adapted assessment should be integrated into multidisciplinary care. QoL evidence remains limited to XLMTM and SELENON-related myopathy. Prospective, genotype-stratified studies including longitudinal, academic, adaptive and caregiver outcomes are needed, particularly alongside emerging disease-modifying therapies.

**Systematic review registration:**

https://www.crd.york.ac.uk/PROSPERO/view/CRD420261373780, identifier CRD420261373780.

## 1 Introduction

Congenital myopathies (CMs) are a clinically and genetically heterogeneous group of rare or ultra-rare inherited muscle disorders typically presenting at birth or in early infancy with hypotonia, proximal weakness and a non-progressive or a variably progressive course (1, 2). Clinical presentations range from severe neonatal forms with respiratory failure to milder, late-onset phenotypes, multidisciplinary management is required including neurological, respiratory, rehabilitative, cardiological and psychosocial care (3–5).

Advances in next-generation sequencing have identified more than 30 causative genes, shifting the diagnostic paradigm from a histopathology-first to a genetics-first approach, with early broad sequencing and selective use of muscle biopsy (2, 6). Despite these developments, CMs are a well-represented group among inherited myopathies, with a reported prevalence of 3.9 per 100,000 and cumulative incidence of 12.3 per 100,000 by age 35 (7).

Clinical management has historically prioritized motor and respiratory outcomes, particularly in severe neonatal presentations characterized by ventilatory dependence and musculoskeletal complications. This has contributed to limited systematic investigation of neuropsychological and psychosocial outcomes, which remain infrequently assessed and largely absent from diagnostic and therapeutic intervention frameworks. Consequently, the extent to which cognitive development, language acquisition, adaptive functioning and psychological well-being are affected across CM subtypes remains unclear, despite direct implications for educational planning, rehabilitation and emerging disease-modifying therapies. Accordingly, the ASPIRO gene therapy trial in X-linked myotubular myopathy, which showed substantial motor and respiratory improvements alongside significant hepatic safety concerns, highlights the need for comprehensive baseline and longitudinal assessment across all outcome domains, including neuropsychological functioning (8).

Furthermore, biological evidence supporting concomitant central nervous system (CNS) involvement provides a strong rationale for investigating neuropsychological outcomes in CMs. Proteins implicated in CM pathogenesis, including myotubularin (*MTM1*), amphiphysin 2 (*BIN1*) and dynamin 2 (*DNM2*), regulate metabolism, membrane remodeling, and vesicle trafficking in both muscle and neuronal systems, contributing to T-tubule formation, synaptic vesicle recycling, and ER–plasma membrane junction organization (9, 10). Similarly, alpha-actin (*ACTA1*), essential for sarcomere assembly, is involved in neuronal cytoskeletal dynamics, including growth cone guidance and dendritic spine formation (11, 12). These shared molecular pathways reflect conserved mechanisms across excitable tissues rather than functional analogies. Moreover, several CM-related genes are expressed in the brain: *MTM1* regulates lysosomal trafficking and *mTOR* signaling relevant to synaptic function (13); *BIN1* produces brain-specific isoforms implicated in endocytosis and tau-related processes (14); and *ACTA1* is expressed across multiple brain regions during development (15, 16). In addition, secondary mechanisms – such as chronic hypoxia, prolonged neonatal intensive care exposure, and restricted motor-driven environmental interaction – may further contribute to neurodevelopmental vulnerability (17).

Together, these factors support the hypothesis that neuropsychological involvement represents an intrinsic and clinically relevant component of CMs. Despite this biological rationale, empirical evidence remains sparse and heterogeneous. Neuropsychological deficits have been previously described in other rare muscular diseases (e.g., Duchenne Muscular Dystrophies and Myotonic Dystrophies), including impairments in intellectual functioning, executive processes, memory, and visuospatial abilities (18), but systematic investigation in CMs is inadequate. Available studies are typically small, methodologically heterogeneous and rarely designed with neuropsychological outcomes as primary endpoints, as well as no standardized assessment framework has been established.

This evidence gap limits the identification of at-risk patients, the integration of cognitive monitoring into clinical care, and the inclusion of neuropsychological outcomes endpoints in therapeutic trials.

This systematic review aims to address this gap by synthesizing evidence on neuropsychological, cognitive, psychosocial, and QoL outcomes in children and adolescents with CMs, examining variability across genetic subtypes and clinical severity, and considering caregiver burden. The objective is to provide an evidence-based foundation for integrating neuropsychological assessment into routine clinical management and to define priorities for future research in this underexplored domain.

## 2 Methods

This systematic review was conducted in accordance with established methodological standards for evidence synthesis following the Preferred Reporting Items for Systematic Reviews and Meta-Analyses (PRISMA) (19) guidelines and has been registered in the PROSPERO database (registration number CRD420261373780). The PRISMA checklist is provided in Supplementary Materials 1.

### 2.1 Research question

The research question was formulated according to a Population, Exposure, Comparison, and Outcomes (PECO) framework (20), as reported in Table 1.

**TABLE 1:** PECO framework used to define the research question.

PECO framework	
Population	- Children and adolescents aged 0–18 years with a diagnosis of congenital myopathy
Exposure/Condition	- Congenital myopathy, including different clinical and genetic subtypes; - Formal neuropsychological/psychological evaluation
Comparator	- No formal comparator was required; - When available, comparisons across genetic subtypes, clinical severity and associated brain abnormalities were considered
Outcomes	- Patients: standardized neuropsychological, cognitive, psychosocial, and quality-of-life outcomes; - Caregiver: burden and quality-of-life

Based on this framework, the primary research question was: What is the reported neuropsychological and psychosocial profile of children and adolescents with CMs, and how frequently are impairments reported among assessed patients?

Secondary questions were: (i) whether reported outcomes differ according to CM subtype, genetic findings, clinical severity, and associated brain abnormalities; (ii) which standardized instruments have been used to assess these outcomes; and (iii) what caregiver burden and quality-of-life outcomes have been reported among children and adolescents with CMs and their caregivers.

### 2.2 Eligibility criteria

Eligibility criteria were defined according to the PECO framework. Studies were included if they involved children and adolescents aged 0–18 years with a diagnosis of CMs established on the basis of clinical, neurophysiological, histopathological and/or genetic criteria.

Studies reporting outcomes in caregivers of affected individuals were also considered eligible.

The exposure of interest included standardized neuropsychological, cognitive, psychological and adaptive assessments administered to patients, as well as measures of caregiver burden and quality of life. Outcomes were collected either directly from patients and caregivers or through validated proxy-report instruments (e.g., parent-reported measures). Studies were included regardless of the presence of a comparison group; however, when available, comparisons across genetic subtypes, age groups, and clinical severity levels were considered.

Eligible outcomes included intellectual functioning, neurodevelopmental milestones, domain-specific cognitive functions (e.g., executive functions, memory, attention, language), learning and academic difficulties, behavioral and emotional symptoms, adaptive functioning, and QoL. Additional outcomes of interest included caregiver burden and QoL, as well as associations between neuropsychological profiles and clinical variables and/or genetic findings.

Observational study designs, including cross-sectional, cohort, and case–control studies, as well as case series and single case reports, were included. Studies were excluded if they focused on congenital muscular dystrophies, congenital myasthenic syndromes or acquired myopathies, or if they did not report neuropsychological, cognitive, or psychological outcomes. Reviews, editorials, conference abstracts, and not peer reviewed studies were excluded. Studies including exclusively adult populations or mixed-age samples without separable pediatric data were not considered. Only studies published in English or Italian were included.

### 2.3 Search strategy

A comprehensive literature search was conducted on June 2026, across PubMed (MEDLINE), Scopus, Web of Science, and ClinicalTrials.gov. No date restrictions were applied.

The search strategy combined terms related to CMs with terms capturing neuropsychological, cognitive, psychological, and quality-of-life outcomes. The full primary search string was as follows: (“congenital myopathy” OR “congenital myopathies” OR “nemaline myopathy” OR “central core disease” OR “multiminicore disease” OR “centronuclear myopathy” OR “myotubular myopathy”) AND (“cognition” OR “cognitive” OR “neuropsychology” OR “neuropsychological” OR “psychological” OR “executive function” OR “attention” OR “memory” OR “learning” OR “language” OR “neurocognitive” OR “IQ” OR “intellectual” OR “quality of life” OR “QoL” OR “well-being” OR “emotional” OR “behavior” OR “social*” OR “caregiver” OR “communic*”) AND (child OR children OR pediatric OR pediatric OR adolescent).

In addition to database searches, the reference lists of all included studies were manually screened to identify further relevant studies. The database-specific search strategies, along with any applied filters, are detailed in the Supplementary Materials 2 – Supplementary Table 1.

### 2.4 Study selection

All records identified through the search strategy were imported into a reference management system, and duplicate entries were removed manually prior to screening. Two reviewers (S.R., G.A.) independently screened titles and abstracts to identify potentially eligible studies. The full texts of all records considered potentially relevant were subsequently retrieved and assessed independently by the same two reviewers according to the predefined eligibility criteria.

Disagreements arising during the screening process or full-text assessment were resolved through discussion with a third reviewer (A.S.). All identified records were managed using the reference management software Zotero (version 7.0.32).

### 2.5 Data extraction

Data extraction was conducted independently by two reviewers (S.R., G.A.) using a standardized form developed specifically for this review. Extracted information included study characteristics (author, year of publication, and country), clinical features (type of CM, genetic/clinical findings, neurodevelopmental milestones), and sample characteristics, including whether data were obtained directly from patients or reported by caregivers, sample size, age, sex distribution, and available socioeconomic information.

Additional data included the neuropsychological instruments used, and outcomes across cognitive, psychological, adaptive, and QoL domains. Information regarding caregiver burden and QoL was also collected.

Clinical severity was extracted according to the classifications reported by the authors of the original studies.

Following independent extraction, the two reviewers compared the extracted data and resolved discrepancies through discussion. When necessary, disagreements were adjudicated by a third reviewer (A.S.) to ensure accuracy and consistency.

### 2.6 Risk of bias and quality of studies assessment

The overall quality and strength of the evidence were evaluated using the Oxford Centre for Evidence-Based Medicine levels of evidence (OCEBM) (21). Risk of bias was assessed using the Joanna Briggs Institute (JBI) critical appraisal tools (22), with design-specific checklists applied according to the study type. Two reviewers (S.R., G.A.) independently assessed the included studies, disagreements were solved through discussion.

### 2.7 Data synthesis

Given the marked heterogeneity in study design, sample characteristics, genetic subtypes, assessment tools, and outcome measures, a quantitative meta-analysis was not considered appropriate. Therefore, a narrative and descriptive synthesis of the findings was performed.

Results were organized into specific developmental and neuropsychological domains, including global cognitive development, intellectual functioning, language development, neuropsychiatric features, and social/adaptive functioning and QoL. Additional domains, such as school-related outcomes, were described when available. Caregiver-related outcomes were analyzed separately.

Descriptive information on population characteristics, clinical features, and genetic findings was also summarized to contextualize the neuropsychological results. Data were reported using descriptive statistics, primarily frequencies and percentages based on available cases within each domain. Where possible, findings were further detailed at the study level, reporting sample size, genetic subtype, and relevant scores. Differences across genetic subtypes and clinical presentations were explored qualitatively, without formal comparative analysis, due to the limited and heterogeneous nature of the available data.

Because the included studies employed instruments with substantially different psychometric properties, findings within each domain are reported separately according to instrument type: (i) parent-report or first-level developmental screeners, such as the Ages and Stages Questionnaire (ASQ) and the Gesell Developmental Schedules (GDS); (ii) clinician-administered developmental scales yielding a developmental quotient, including the Griffiths III, Bayley III, and Illinois Test of Psycholinguistic Abilities (ITPA); and (iii) standardized intelligence tests yielding an IQ (Wechsler scales, Raven’s Colored Progressive Matrices). Two studies that reported a numerical score without specifying the instrument were assigned to a category on the basis of the score reported (developmental quotient or IQ).

## 3 Results

**FIGURE 1:**
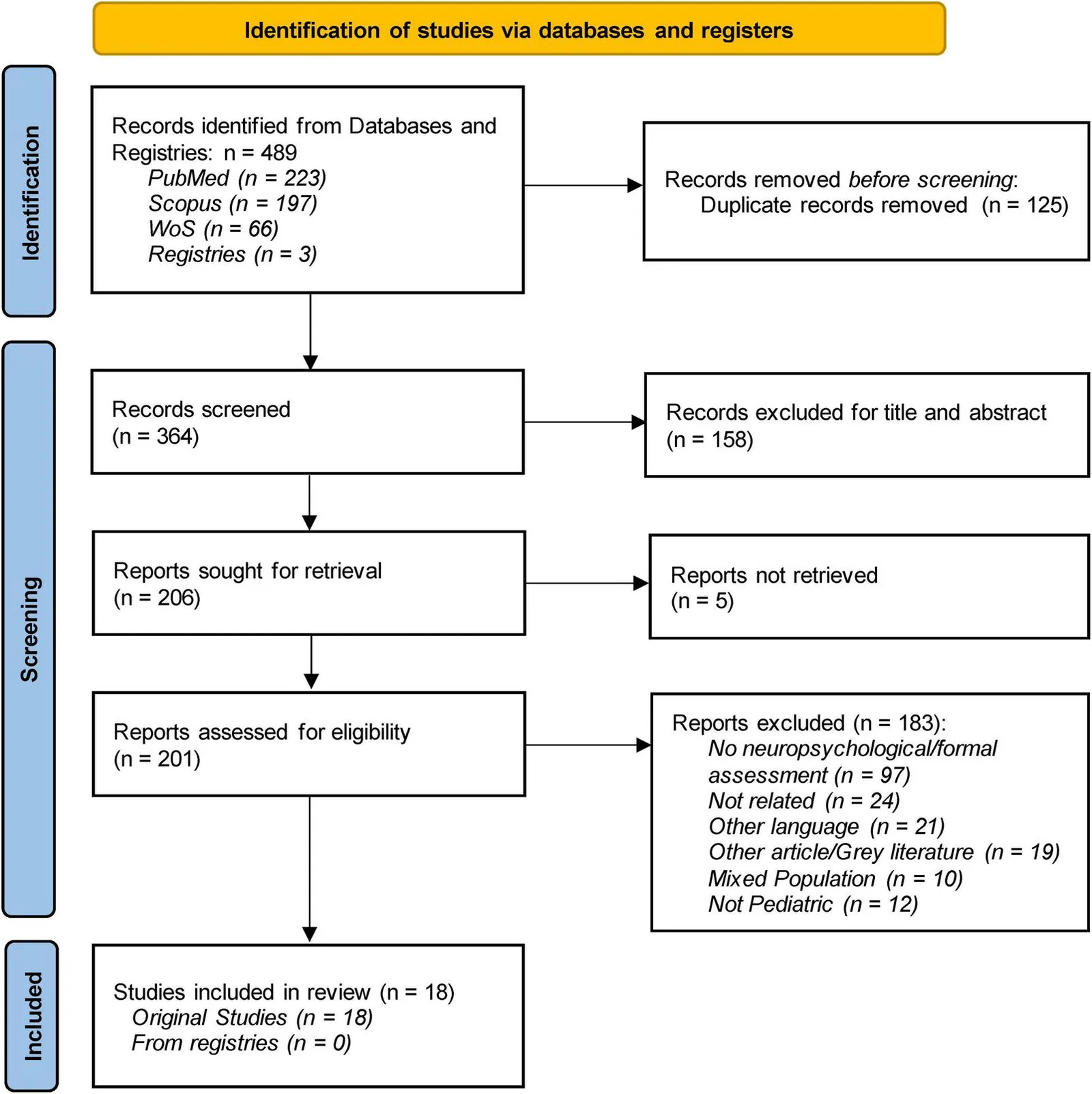
PRISMA flow diagram of the study selection process.

The study selection process is summarized in Figure 1. A total of 489 studies were retrieved; after duplicate removal and the screening process, a total of 18 studies met the inclusion criteria and were included in the review (23–40). Among the included studies, 15 examined patient outcomes, two focused on caregiver outcomes (28, 31) and one addressed both populations (27).

### 3.1 Study design and primary outcome of interest

The overall body of evidence was characterized by a predominance of small-scale and descriptive designs. Most studies were case reports or case series (*N* = 13/18, 72.2%), alongside three cross-sectional studies, one prospective cohort study, and one qualitative study.

Only a minority of studies (*N* = 5/18, 27.8%) primarily focused on neuropsychological or cognitive developmental outcomes and/or caregiver burden and QoL. In the remaining studies, these aspects were reported as secondary findings within broader clinical, genetic, or neuromuscular investigations.

### 3.2 Risk of bias and quality assessment

According to the OCEBM levels of evidence, most included studies provided low-level evidence (Level 4; 14/18, 77.8%), while only four studies (22.2%) were classified as Level 2. Consistently, the risk of bias assessment showed that 8/18 studies (44.4%) were rated as low risk, 6/18 (33.3%) as moderate risk, and 4/18 (22.2%) as high risk (Table 2).

**TABLE 2:** Risk of Bias summary.

Author, Reference	Design	N. of applicable items	% Yes	Overall RoB
Alkhunaizi et al. (23)	Case series	3/9	33%	**High**
Barreto-Mota et al. (24)	Case report	4/7	57%	**Moderate**
Bouman et al. (40)	Cross-sectional	6/8	75%	**Low**
Buchignani et al. (25)	Case report	4/7	57%	**Moderate**
Cumbo et al. (26)	Cross-sectional	6/8	75%	**Low**
Dowling et al. (27)	Cohort study	5/8	62%	**Moderate**
Duong et al. (28)	Cross-sectional	5/7	71%	**Low**
Herman et al. (29)	Case series	5/10	50%	**Moderate**
Kouwenberg et al. (30)	Case report	4/5	80%	**Low**
Merchán Arjona et al. (31)	Qualitative	10/10	100%	**Low**
Mohamadian et al. (39)	Case report	5/7	71%	**Low**
Nóbrega et al. (32)	Case series	3/9	33%	**High**
PeBenito et al. (33)	Case report	7/7	100%	**Low**
Saito et al. (34)	Case series	2/9	22%	**High**
Wallgren-Pettersson et al. (35)	Case series	8/10	80%	**Low**
Wang et al. (36)	Case report	2/5	40%	**High**
Yildirim et al. (37)	Case report	3/6	50%	**Moderate**
Zhang et al. (38)	Case series	6/9	67%	**Moderate**

JBI checklists applied according to study design (22). Overall judgment on applicable items: Low risk ≥ 70% Yes; Moderate risk 50–69%; High risk < 50%. RoB: Risk of Bias.

### 3.3 Population

Across the included studies, a total of 104 pediatric patients with CMs were identified (*N* = 92 males, 88.5%; *N* = 12 females, 11.5%). In addition, data on 56 caregivers were included (*N* = 8 males, 14.3%; *N* = 14 females, 25.0%; *N* = 34 gender not reported, 60.7%). Age at evaluation showed substantial variability across studies. Individual-level data were available for 49/104 (47.1%) patients, with ages ranging from 0.75 to 16 years (median = 7 years; IQR = 6.3). For the remaining 55 patients (52.9%), age was reported at the study level, with mean or median values spanning from early infancy to adolescence. Specifically, one study (*n* = 9) reported a mean age of 6.49 years (SD = 4.51), another (*N* = 34) a median age of 1.2 years (range 0.3–4.6), and a third (*N* = 12) a mean age of 16 years, with no SD provided.

Caregiver age was reported only for a small subset of participants (*N* = 5/56, 8.9%), ranging from 28 to 44 years, whereas no age information was available for the remaining caregivers.

### 3.4 Age at onset and diagnosis

Age at symptom onset, when reported, ranged from birth to 5 years. Among the 54/104 (51.9%) patients for whom this information was available, most (*N* = 51/54, 94.4%) presented within the first year of life, while only a small number of cases (*N* = 3/54, 5.6%) had onset after 1 year of age.

Age at diagnosis was reported in 50/104 (48.1%) patients, showing considerable variability, ranging from the prenatal period (−0.2 years) to 12 years. The time interval between symptom onset and diagnosis could not be determined due to the lack of individual-level data and inconsistent reporting across studies.

### 3.5 Myopathy subtypes and clinical severity

X-linked myotubular myopathy (XLMTM) was the most frequently reported subtype, accounting for 74 out of 104 patients (71.2%). Nemaline myopathy was identified in 15 cases (14.4%), followed by selenoprotein N-related myopathy (*N* = 8, 7.7 %), centronuclear myopathy (*N* = 3, 2.9%), CM associated with *ACTA1* mutations (*N* = 3, 2.9%), and *RYR1*-related CM (*N* = 1, 0.9%).

Among caregivers, most were caring for individuals with XLMTM (*N* = 39/56, 69.6%), while the remaining were associated with nemaline myopathy (*N* = 17, 30.4%).

Severity distribution, as classified in the original studies by the authors, varied across the included patient cohort, with the majority of patients classified as severe (*N* = 60/104, 57.7%), followed by mild (*N* = 19/104, 18.3%), moderate (*N* = 15/104, 14.4%), and moderate-to-severe (*N* = 3/104, 2.9%) forms, while severity was not explicitly classified for 7 patients (6.7%). XLMTM (*MTM1*) accounted for the largest proportion of severe cases (*N* = 55/60, 91.7%), whereas other subtypes, including nemaline and centronuclear myopathies, were more frequently represented in the mild-to-moderate range, albeit representing a smaller proportion of the overall cohort.

### 3.6 Genetic findings

**FIGURE 2:**
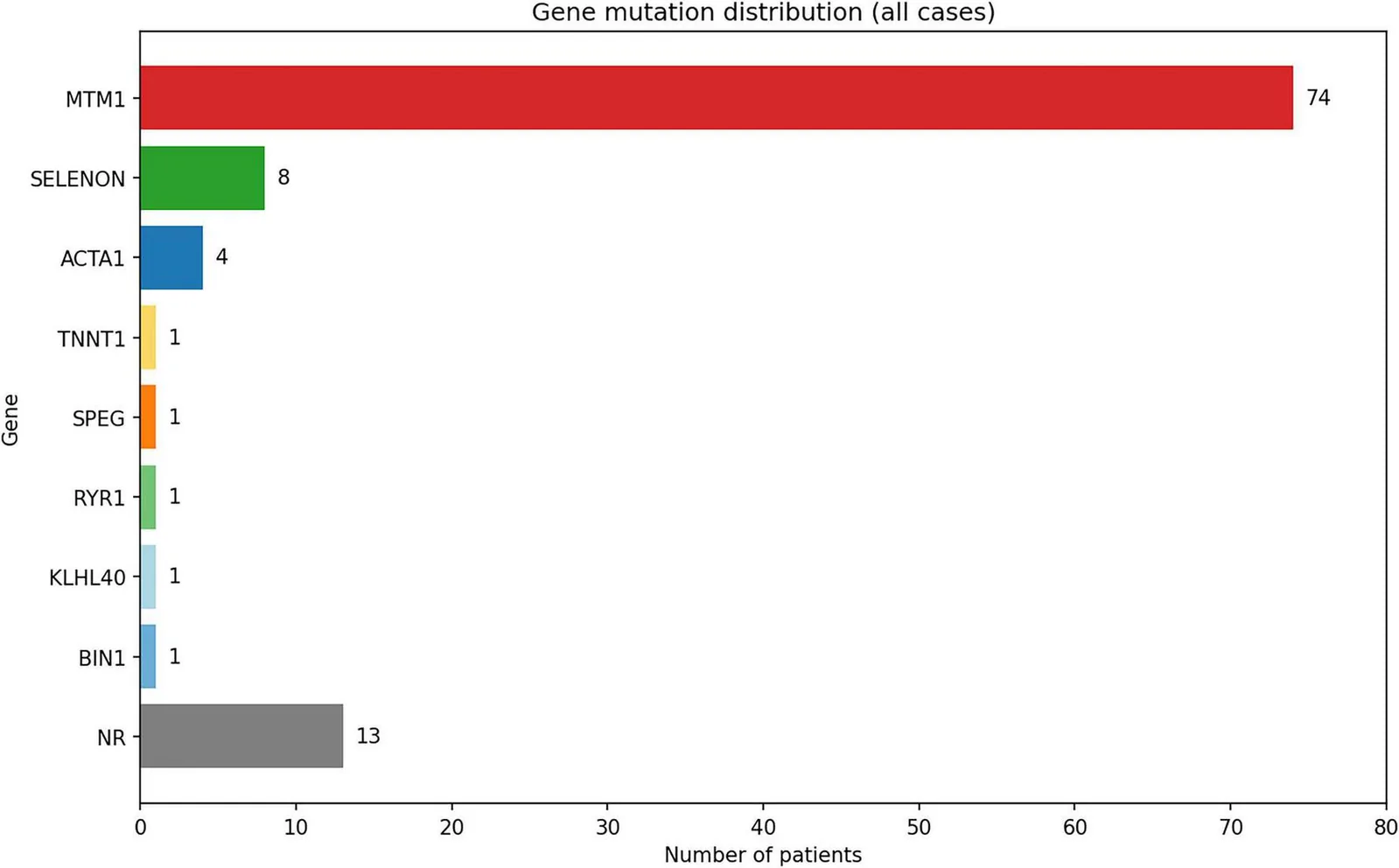
Distribution of identified gene mutations in patients with congenital myopathies. NR: not reported.

As Figure 2 shows, genetic data were available for most of the included patients (*N* = 91/104, 87.5%) and revealed a marked predominance of MTM1 mutations, accounting for 74/91 cases (81.3%). The remaining cases were distributed across multiple genes, including SELENON (*N* = 8, 8.8%), ACTA1 (*N* = 4, 4.4%) and single cases involving TNNT1, SPEG, RYR1, KLHL40 and BIN1.

Among *MTM1* cases, missense (28.38%) and nonsense (25.68%) variants were most frequent, followed by frameshift (16.22%) and deletions (13.51%). In contrast, non-*MTM1* genotypes showed a more heterogeneous pattern, with *ACTA1* variants predominantly missense and isolated reports of compound heterozygous and splicing variants in other genes. Detailed variant-level data are reported in Supplementary Materials 2- Supplementary Table 2.

### 3.7 Clinical features

Key clinical features were analyzed to contextualize neuropsychological outcomes and their potential impact on adaptive functioning, QoL, and caregiver burden.

#### 3.7.1 Respiratory and feeding outcomes

Respiratory support data were reported in 74 out of 104 patients (71.2%). Among them, invasive ventilation via tracheostomy was the most frequently reported modality (*N* = 35/74, 47.3%), followed by non-invasive ventilation (NIV) (*N* = 15/74, 20.3%). A smaller proportion of patients did not require respiratory support (*N* = 22/74, 29.7%), while unspecified mechanical support was reported in two cases (*N* = 2/74, 2.7%).

Feeding data were available for 65/104 patients (62.5%), where tube feeding (e.g., PEG) was the most common modality (*N* = 45/65, 69.2%), whereas no difficulties were reported in 18/65 patients (27.7%). Only isolated cases of feeding difficulties without support (*N* = 1/65, 1.5%) and partial oral feeding with PEG support (*N* = 1/65, 1.5%) were described.

#### 3.7.2 Neurological and motor findings

Neurological findings were variably reported across studies. Hypotonia and muscle weakness were the most frequently described features (*N* = 35/40, 87.5% and *N* = 49/60, 81.7%, respectively). Ophthalmoplegia and/or ptosis were reported in over half of cases (*N* = 20/33, 60.6%), while facial weakness (*N* = 28/56, 50.0%) and abnormal reflexes (*N* = 12/41, 29.3%) were observed in a smaller proportion of patients. Moreover, seizures were also reported in a minority of patients (*N* = 5/31, 16.1%) among those with available data.

Delayed motor milestone acquisition was reported in nearly all patients (*N* = 94/98, 95.9%). Where data were available, delayed or impaired walking was reported in 68/73 patients (93.2%), followed by delayed or impaired sitting (*N* = 36/49, 73.5%) and head control (*N* = 6/40, 15.0%).

#### 3.7.3 Associated brain abnormalities

Brain imaging data were available in a subset of patients (*N* = 50/104, 48.1%), with normal findings reported in the majority of cases (*N* = 44/50, 88.0%), whereas structural abnormalities, when present, were heterogeneous. Detailed study-level findings are reported in Table 3.

**TABLE 3:** Summary of clinical and neuropsychological findings.

Study	Study design	Subjects (*N* = 104)	Age at evaluation	Type of CM	Associated brain abnormalities	Outcome measures	Score/outcome	Summary of findings
Alkhunaizi et al. (23)	Case series	1	13 months	*RYR1*-related congenital myopathy (recessive)	Normal brain MRI	ASQ	Normal ASQ-scores for communication, fine motor, problem-solving, personal-social; gross motor delayed	Gross motor significantly delayed, while other developmental domains were within normal range.
Barreto-Mota et al. (24)	Case report	1	4 years	X-linked Myotubular Myopathy (Centronuclear myopathy)	Brachycephaly; enlargement of subarachnoid spaces in frontal-parietal regions; reduction of parenchymal volume; wide posterior fossa with rotation of vermis; arachnoid cyst in left cerebellar hemisphere.	Formal assessment (instrument not specified)	Global developmental quotient = 70	Mild XLMTM presenting with global developmental delay (DQ = 70), expressive language disorder, and sensory processing disorder. Significant improvement with therapies. Mild motor delay. No cognitive impairment explicitly reported..
Bouman et al. (40)	Cross-sectional	6	Patient 1: 16y Patient 2: 3y Patient 3: 8y Patient 4: 9y Patient 5: 13y Patient 6: 11y	Selenoprotein N-related Myopathy (*SEPN1*-RM)	n.r.	PedsQL-GC + PedsQL-NM	1) PedsQL-GC: - self-report: 55.7 ± 15.1 - Proxy-mother: 52.4 ± 8.4 - Proxy-father: 52.9 ± 10.0 2) PedsQL-NM - self-report: 61,8 ± 11,4 - Proxy-mother: 57.7 ± 10.1 - Proxy-father: 53.8 ± 13.8	Reduced generic quality of life across self- and parent-proxy reports; neuromuscular-specific QoL scores were comparable to those reported in SMA.
Buchignani et al. (25)	Case report	1	9 months	*KLHL40*-related Nemaline Myopathy (NEM8)	n.r.	Bayley III	Bayley III DQ at 37th percentile	At 9 months age, DQ score in the low normal range for age. Motor function mildly improved over 12-year follow-up. Language delayed but improved with therapy. Currently cognitive abilities preserved.
Cumbo et al. (26)	Cross-sectional	9	Mean 6.49 years (SD 4.51)	X-linked Myotubular Myopathy (XLMTM)	n.r.	Griffiths III (*n* = 4); Raven’s Coloured Progressive Matrices (*n* = 4); VABS-II (*n* = 9); CARS2-ST (*n* = 9); CY-BOCS (*n* = 5)	Griffiths III: mean DQ = 79.3 (GMDS-A = 89.3; GMDS-B = 83.3; GMDS-C = 86.5; GMDS-D = 88.3; GMDS-E = 61.8.); RCPM median percentile = 30.5 (range 2-93); VABS-II mean Adaptive IQ = 52.4; CARS2-ST mean T = 31.89; CY-BOCS mean = 7	Neurocognitive profile within lower limits of the norm. Borderline scores in language. Gross motor severely impaired. Global adaptive deficit. Perseverative behavioural traits present but subclinical for ASD and OCD.
Dowling et al. (27)	Cohort study	34	Median 1.2 years (range 0.3–4.6)	X-linked Myotubular Myopathy (XLMTM)	n.r.	PedsQL-NM	PedsQL-NM mean = 50.3	Moderately reduced quality of life
Herman et al. (29)	Case series (retrospective)	30	Median = 4; Range = 1.8–13	X-linked Myotubular Myopathy (XLMTM)	1/30: Dandy-Walker malformation; progressive communicating hydrocephalus requiring VP shunt. 4/30: seizures (not specified)	Formal assessment (instrument not specified)	Cognitive development delayed in 6/30 and normal in 24/30.	Cognitive development was delayed in a minority of patients (20.0%), while the majority showed age-appropriate cognitive functioning (80.0%).
Kouwenberg et al. (30)	Case report	1	8 years	Autosomal Dominant Centronuclear Myopathy (*BIN1*-related)	n.r.	WPPSI-III-NL	Total IQ = 77; VIQ = 89; PIQ = 67; PSQ = 85; GLC = 91	Reduced IQ (borderline). Disharmonic profile with verbal IQ higher than performance IQ. Processing speed just below average. General language composite normal.
Mohamadian et al. (39)	Case report	1	9 years	Selenoprotein N-related Myopathy (*SEPN1*-RM / RSMD1)	n.r.	Formal assessment (instrument not specified)	IQ = 48 (moderate intellectual disability)	Moderate intellectual disability, with delayed motor milestones and dysarthria.
Nóbrega et al. (32)	Case series	1	7 years	Nemaline Myopathy (*ACTA1*-related)	Mild cerebral atrophy with marked atrophy of the posterior half of the corpus callosum	MoCA; CARS	MoCA = 21/30; CARS = 41/60	Severe ASD and deficits in various cognitive domains. Dependent for basic activities of daily living.
PeBenito et al. (33)	Case report	1	7.5 years	Centronuclear Myopathy (no molecular diagnosis)	EEG: right inferior frontal spike discharge + generalized polyspike discharges.	WISC-R	Full IQ = 106; Verbal IQ = 114; Performance IQ = 96	Normal intelligence; VIQ > PIQ pattern.
Saito et al. (34)	Case series	3	Patient 1: 5y5m; Patient 2: 2y4m; Patient 3: 4y	Congenital Myopathy due to *ACTA1* mutations (severe infantile type).	Decreased cerebral white matter volume, enlargement of lateral ventricles (anterior horn predominance), frontal lobe hypoplasia in all patients. Patient 3: posterior fossa arachnoid cyst.	ITPA (patient 1); KIDS (patient 2 and 3)	ITPA age-level: 3y6m; KIDS word comprehension at 9 and 10 months	All 3 patients showed global developmental delay and limited word comprehension during early childhood.
Wallgren-Pettersson et al. (35)	Case series	12	Mean 16 years	Congenital Nemaline Myopathy	Normal CT head and normal EEG normal in all patients	Wechsler intelligence tests; Beery VMI; Sentence-completion test	IQ range 89-136; mean 107.9	Normal IQ across patients, with strengths in spatial perception and arithmetic; some auditory short-term memory weaknesses; positive self-concept and good communicative abilities.
Wang et al. (36)	Case report	1	15 months	*TNNT1*-related Nemaline Myopathy	n.r.	Gesell Development Schedules	Moderate gross motor delay; mild language delay; personal-social borderline	Moderate gross motor delay, mild language delay, and borderline personal-social development.
Yildirim et al. (37)	Case report	1	7 years	Centronuclear Myopathy (*SPEG*-related)	Normal brain MRI	Formal assessment (instrument not specified)	IQ = 55 (mild intellectual disability)	Mild intellectual disability (IQ = 55); motor delay, social and language skills mildly delayed.
Zhang et al. (38)	Case series	1	14 years	Selenoprotein N-related Myopathy (*SEPN1*-RM / multiminicore disease)	Normal brain MRI	Wechsler IQ test	IQ = 45 (moderate intellectual disability)	Moderate intellectual disability with poor learning performance.

ASQ: Ages and Stages Questionnaire; ASD: Autism Spectrum Disorder; Bayley III: Bayley Scales of Infant and Toddler Development, Third Edition; CARS/CARS2-ST: Childhood Autism Rating Scale (Second Edition – Standard Version); CM: Congenital Myopathy; CT: Computed Tomography; CY-BOCS: Children’s Yale-Brown Obsessive Compulsive Scale; DQ: Developmental Quotient; EEG: Electroencephalography; GLC: General Language Composite; GMDS: Griffiths Mental Development Scales; IQ: Intelligence Quotient; ITPA: Illinois Test of Psycholinguistic Abilities; KIDS: Kyoto Scale of Psychological Development; MoCA: Montreal Cognitive Assessment; MRI: Magnetic Resonance Imaging; NEM8: Nemaline Myopathy type 8; N.R.: Not Reported; OCD: Obsessive-Compulsive Disorder; PedsQL-GC: Pediatric Quality of Life Inventory – Generic Core Scale; PedsQL-NM: Pediatric Quality of Life Inventory – Neuromuscular Module; PIQ: Performance Intelligence Quotient; PSQ: Processing Speed Quotient; RCPM: Raven’s Coloured Progressive Matrices; SD: Standard Deviation; SEPN1-RM: Selenoprotein N-related Myopathy; SMA: spinal muscular atrophy; VABS-II: Vineland Adaptive Behavior Scales, Second Edition; VIQ: Verbal Intelligence Quotient; VP: ventriculoperitoneal; WISC-R: Wechsler Intelligence Scale for Children – Revised; WPPSI-III-NL: Wechsler Preschool and Primary Scale of Intelligence, Third Edition (Dutch version); XLMTM: X-linked Myotubular Myopathy.

### 3.8 Global development and neuropsychological outcomes

#### 3.8.1 Outcome measures

Outcome measures were heterogeneous across studies and spanned multiple domains. Global developmental assessment was most commonly performed using the Griffiths Mental Development Scales (*N* = 4/104, 3.8%), while other tools were only occasionally used, including the Kinder Infant Development Scale (KIDS; *N* = 2/104, 1.9%), the Bayley Scales of Infant and Toddler Development (*N* = 1/104, 1.0%), the ASQ (*N* = 1/104, 1.0%), and the GDS (*N* = 1/104, 1.0%). Assessment of intellectual functioning was less frequent and based on Wechsler scales (*N* = 15/104, 14.4%) and Raven’s Colored Progressive Matrices (RCPM; *N* = 4/104, 3.8%).

Specific neuropsychological domains were primarily assessed using the Beery Visual-Motor Integration test (*N* = 12/104, 11.5%) and the sentence-completion tasks (*N* = 12/104, 11.5%), followed by the ITPA (*N* = 1/104, 1.0%) and the Montreal Cognitive Assessment test (MoCA) (*N* = 1/104, 1.0%). Adaptive functioning was evaluated using the Vineland Adaptive Behavior Scales-II edition (VABS-II; *N* = 9/104, 8.7%), while behavioral and neuropsychiatric features were mainly assessed with the Childhood Autism Rating Scale (CARS; *N* = 10/104, 9.6%) and the Children’s Yale-Brown Obsessive Compulsive Scale (CY-BOCS; *N* = 5/104, 4.8%). Finally, QoL was assessed using the PedsQL-NM/GC in 40/104 children (38.5%).

#### 3.8.2 Overall neuropsychological outcomes

Neuropsychological and cognitive findings were variably reported across studies and are summarized in Table 3.

Data on cognitive functioning across domains were available for 64 out of 104 patients (61.5%). Among these, functioning was within the expected range for age in 39/64 patients (60.9%), whereas specific cognitive impairment or global developmental delay was described in 25/64 patients (39.1%). Out of 104 included subjects, adaptive behavioral difficulties (*N* = 9/104, 8.7%) and neuropsychiatric symptoms (*N* = 10/104, 9.6%) were reported in a smaller subset of patients.

Finally, quality of life outcomes were reported in 40/104 patients (38.5%), all indicating a moderate decrease in QoL.

**FIGURE 3:**
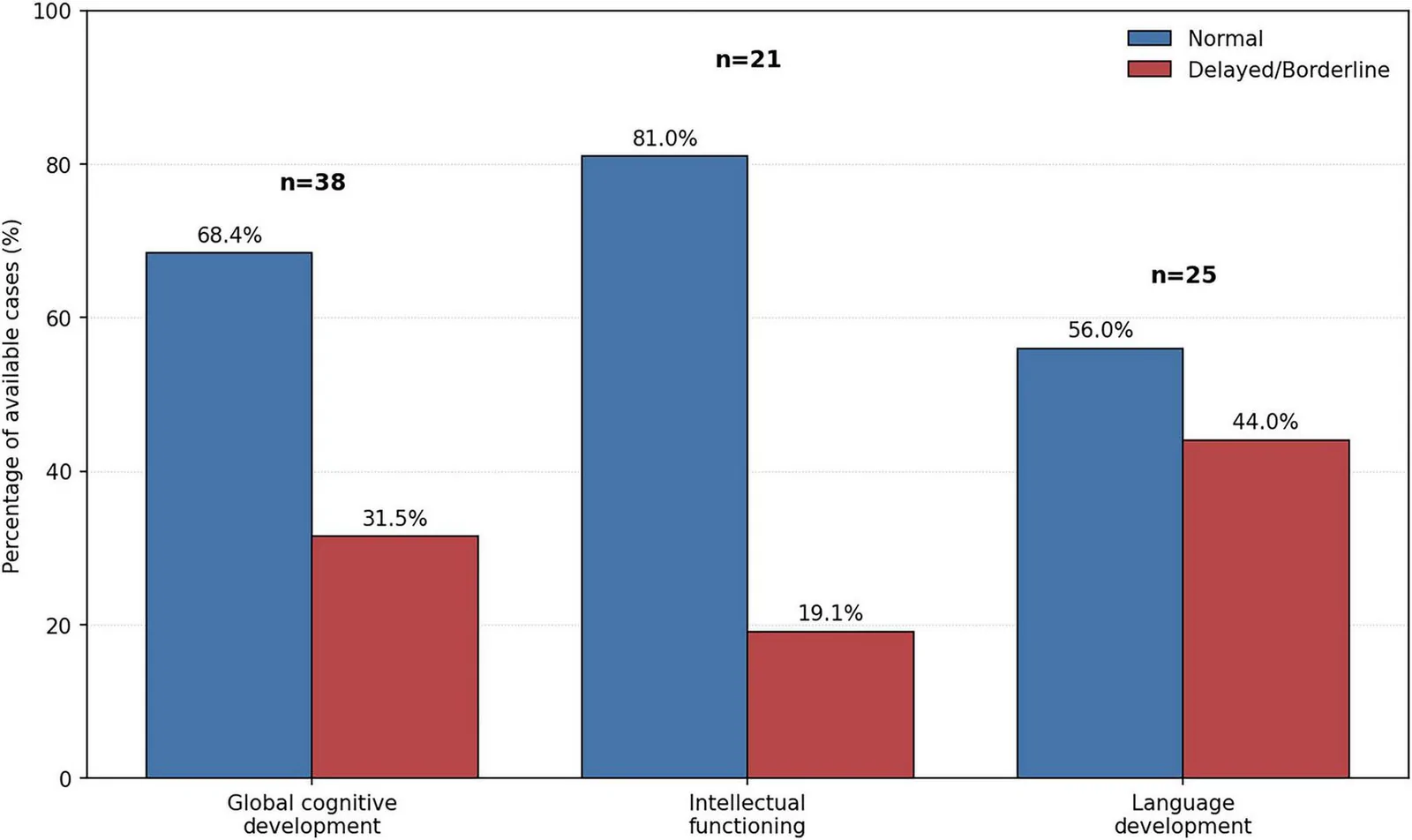
Cognitive outcomes across developmental domains. Bar plots show the proportion of patients classified as normal or delayed/borderline across global cognitive development, intellectual functioning, and language development. Percentages are calculated based on available cases per domain (N indicated above each group).

The distribution of cognitive outcomes across developmental domains is illustrated in Figure 3.

#### 3.8.3 Global cognitive development

Data on global cognitive development were available for 38 out of 104 patients (36.5%) (Figure 3). Among these, development was reported as normal in 26/38 patients (68.4%), delayed in 11/38 (28.9%), and borderline in 1/38 (2.6%). Stratified by instrument type, only one patient was assessed using parent-report or first-level screening tools (ASQ), yielding a normal outcome. The remaining 37/38 underwent clinician-administered developmental testing or an unspecified developmental quotient assessment. However, 30 of these 37 patients came from a single cohort using an unspecified assessment, limiting the robustness of the pooled estimate.

#### 3.8.4 Intellectual functioning

**FIGURE 4:**
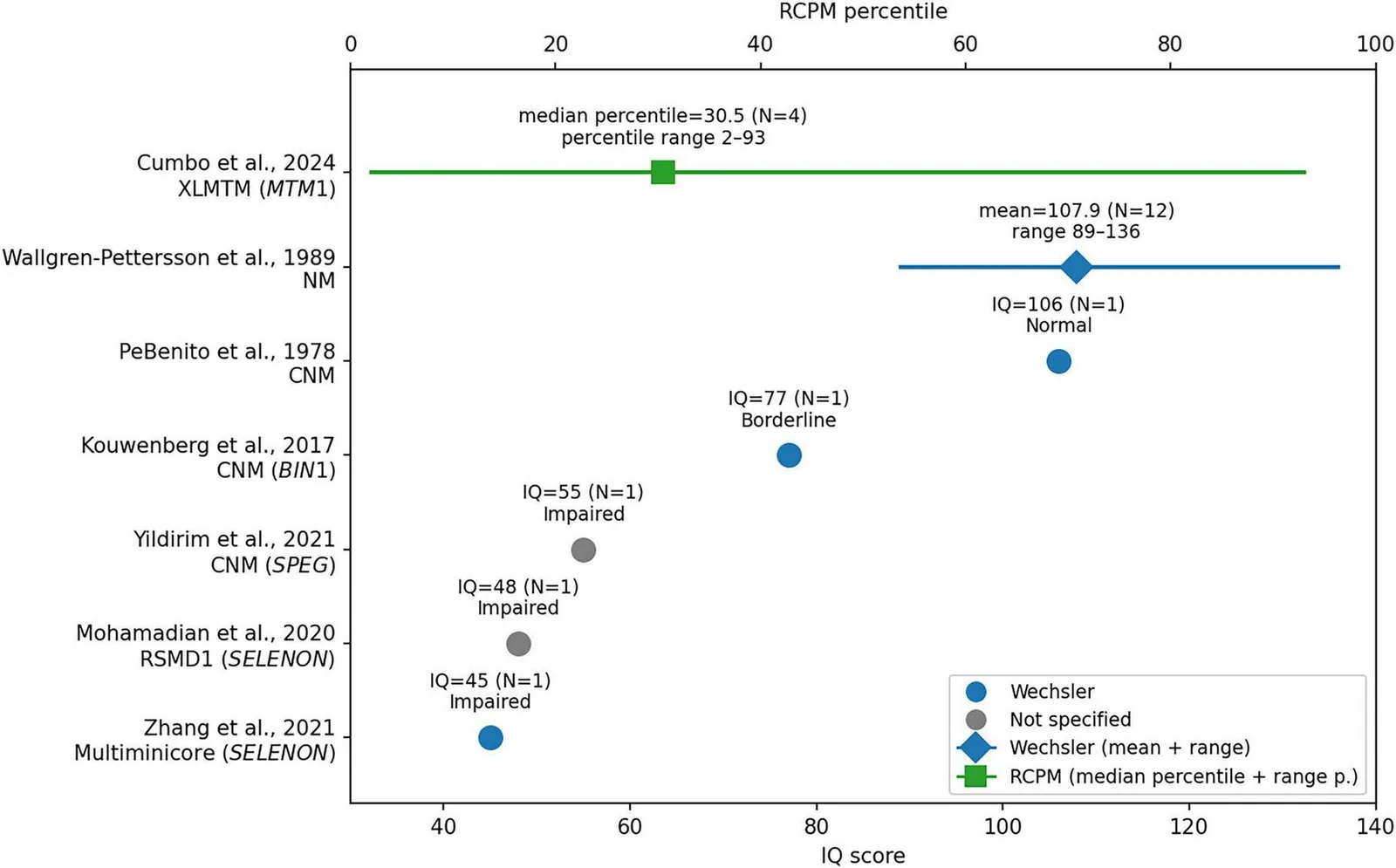
Intellectual functioning across studies. Individual IQ scores are shown as single data points, color-coded by instrument: Wechsler scales (blue) Raven coloured progressive matrices (RCPM; green), and unspecified tools (grey). Wallgren-Pettersson et al. (35) report a group mean (diamond; mean = 107.9; range: 89–136; *n* = 12). Cumbo et al. (26) report RCPM raw scores on the upper axis (square; midpoint = 47.5; range: 2–93; *n* = 4). Type of congenital myopathy and genetic findings are reported for each study, with the implicated gene in parentheses. NM: Nemaline Myopathy; CNM: Centronuclear Myopathy; RSMD1: Rigid-Spine Muscular Dystrophy 1. No gene is reported in Wallgren-Pettersson et al. and PeBenito et al. (33, 35), as these studies predate molecular genetics.

Intellectual functioning was assessed in 21 of 104 patients (20.2%) (Figure 3). Among these, intellectual functioning was reported as normal in the majority of cases (*N* = 17/21, 81.0%), while borderline intellectual functioning was described in 1/21 patients (4.8%) and intellectual disability in 3/21 (14.3%) (Figure 4). Overall, IQ scores showed a broad variability, ranging from 45 to 136. Unlike the global cognitive development domain, intellectual functioning was assessed using standardized IQ measures in 19/21 patients. The remaining two patients had IQ values derived from unspecified instruments; no parent-report or first-level screening tools were used.

#### 3.8.5 Language development

Language development data were reported for 25 of 104 patients (24.0%) (Figure 3). Of these, 14/25 (56.0%) were within the normal range, 7/25 (28.0%) showed delay, and 4/25 (16.0%) were classified as borderline. Stratified by instrument type, language outcomes were derived from direct assessment in 20/25 patients (e.g., Griffith III; *N* = 13 within normal limits). Parent- or patient-reported instruments (e.g., ASQ, KIDS) were used in 3/25 patients (*N* = 2 delayed), while the remaining 2/25 were classified as delayed based on clinical description without formal language testing.

In particular, language delay was reported across multiple subtypes, including KLHL40- and TNNT1-related nemaline myopathy, SPEG-related centronuclear myopathy, as well as in ACTA1-related CM and XLMTM.

#### 3.8.6 Neuropsychiatric outcomes

Neuropsychiatric findings were assessed in 10 out of 104 patients (9.6%) across two studies. In a cohort of 9 patients with XLMTM, Cumbo et al. (26) reported perseverative behavioral traits, although the clinical threshold for autism spectrum disorder and/or obsessive-compulsive disorder had not been met.

In contrast, Nóbrega et al. (32) described a 7-year-old boy with *ACTA1*-related nemaline myopathy and marked atrophy of the posterior half of the corpus callosum presenting with severe autistic features, associated with deficits across multiple cognitive domains and dependence in basic activities of daily living.

#### 3.8.7 Social functioning, adaptive behavior and QoL

Social functioning, adaptive behavior and QoL were scarcely reported across studies. Specifically, age-appropriate functioning emerged in a limited number of cases. In contrast, impairments in adaptive and social functioning were observed across several subtypes. In patients with XLMTM, Cumbo et al. (26) reported global adaptive deficits, accompanied by reduced personal–social skills. Milder alterations were reported in *TNNT1*-related nemaline myopathy (36), with borderline personal-social development, and in *SPEG*-related centronuclear myopathy (37), with mildly delayed social functioning.

Finally, QoL was evaluated only in patients with XLMTM (27) and *SELENON*-related myopathy (40), with both studies reporting reduced QoL.

#### 3.8.8 School outcomes

School-related data were available for only three patients (*N* = 3/104, 2.9%). Kouwenberg et al. (30) described difficulties in concentration and information processing at school in an 8-year-old girl with autosomal dominant centronuclear myopathy (*BIN1*-related). Nóbrega et al. (32) reported severe learning impairments in a 7-year-old boy with *ACTA1*-related nemaline myopathy, including inability to recognize colors or numbers and absence of reading and writing skills. Similarly, Zhang et al. (38) described poor academic performance in a 14-year-old boy with *SELENON*-related myopathy, with marked difficulties in basic mathematical abilities.

### 3.9 Caregiver burden and QoL

Only three studies reporting caregiver-related outcomes were identified, including 56 caregivers of children with CMs (Table 4). Quantitative data were available for 39/56 caregivers (69.6%), while qualitative data were reported for the remaining 17/56 (30.4%). Overall, findings indicate a substantial caregiving burden, with high levels of required support and variable QoL and emotional well-being.

**TABLE 4:** Caregivers’ burden and QoL findings.

Authors	Study design	N. (Gender)	Age at Evaluation (years)	Patients’ CM type	Score/outcome	Summary of findings
Dowling et al. (27)	Cohort study	34 (n.r.)	n.r.	XLMTM	ACEND: mean = 29.0 ± 12.8	High caregiver support required
Duong et al. (28)	Cross-sectional	5 (4 F; 1 M)	Range: 28–44 (28; 44; 34; 43; 42); [Patients range: <1–7 (<1; 3; 4; 6; 7)]	XLMTM	EQ-5D-5L: No impairment in mobility/self-care (all level 1); moderate–severe limitations in usual activities (levels 2–4); minimal pain (one moderate case); variable anxiety/depression (levels 1–4). EQ-5D-5L index: 0.644–0.864; VAS: 58–85	No limitations in mobility/self-care; moderate–severe limitations in usual activities; low pain; variable anxiety; overall moderate–good QoL with variability
Merchán Arjona et al. (31)	Qualitative study	17 (10 F; 7 M)	n.r.	Nemaline myopathy	n.r. (see Summary of findings)	(Qualitative study) Four themes: emergency management, monitoring deterioration, guiding clinicians, ICU transition; high emotional burden and advanced caregiver clinical role

XLMTM: X-linked myotubular myopathy; ACEND: Assessment of Caregiver Experience with Neuromuscular Disease; EQ-5D-5L: EuroQol 5-Dimension 5-Level questionnaire; VAS: Visual Analog Scale; QoL: Quality of life; NR: Not reported.

Specifically, Dowling et al. (27) evaluated caregiver burden in 34 caregivers with XLMTM using the ACEND scale, showing high levels of required support (mean 29.0, SD 12.8) that remained stable over time. Similarly, Duong et al. (28) assessed five caregivers of patients with XLMTM using the EQ-5D-5L and visual analogue scale, reporting no limitations in mobility and self-care, but moderate to severe impairments in usual activities. Pain/discomfort was generally low, whereas anxiety and depression ranged from none to severe; overall health status was rated as moderate to good.

Finally, Merchán Arjona et al. (31), in a qualitative study involving 17 caregivers of children with nemaline myopathy, identified four main themes, including the role of caregivers as first responders, management of clinical deterioration, guidance of healthcare professionals, and significant emotional burden during intensive care transitions, highlighting the substantial clinical and emotional demands placed on caregivers in this population.

## 4 Discussion

This systematic review represents the first comprehensive synthesis of cognitive and neuropsychological outcomes in pediatric CMs, consolidating evidence from 18 studies encompassing 104 children and adolescents and 56 caregivers. The overarching picture that emerges is one of significant heterogeneity: while many children with CMs demonstrate age-appropriate cognitive development and intellectual functioning, a meaningful subset – particularly those with associated brain abnormalities – presents with global developmental delay, language impairment, adaptive deficits or neuropsychiatric features. These findings extend the traditionally predominant focus on clinical aspects – particularly physical outcomes – and underscore the importance of integrating systematic neuropsychological surveillance into the multidisciplinary management of pediatric CMs.

A key issue emerging from the population studied in this review concerns the extent to which motor and speech impairments, together with sensory deficits, may confound the assessment and interpretation of neurodevelopmental and neuropsychological outcomes in pediatric CMs.

As known, language and speech are distinct constructs: language reflects the organization of phonological, lexical, morphosyntactic, and pragmatic representations, whereas speech denotes the motor production of intelligible vocal output through coordinated articulatory, phonatory, and respiratory processes (41, 42). In pediatric CMs – particularly in phenotypes with bulbar involvement (e.g., XLMTM, severe ACTA1) or long-term ventilatory dependence – this motor substrate is frequently compromised, leading to direct implications for assessment. In XLMTM, hearing impairment is also a common phenotypic feature and may further affect language acquisition and performance on verbally mediated tasks (43). Many standardized developmental and cognitive tools – including language scales, global developmental measures (e.g., Griffiths, Bayley), and intelligence tests (e.g., Wechsler scales) – rely on motor output, response speed, or verbal production. Consequently, performance may reflect motor or expressive constraints rather than true cognitive or linguistic ability, leading to potential underestimation of functioning (44, 45).

Although direct evidence in CMs is limited, neurobiological models add further complexity, indicating partial overlap between language and motor systems within inferior frontal and premotor regions, including Broca’s area (BA44/45) (42, 46, 47). Motor impairment may therefore both constrain performance and interact with neural substrates of language and cognitive development, contributing to the heterogeneous profiles observed.

Accordingly, motor, cognitive, and linguistic dimensions are best conceptualized as partially interacting, underscoring the need for cautious interpretation of these findings.

In light of this heterogeneity, the following sections examine neuropsychological outcomes across specific conditions within the spectrum of CMs, integrating clinical and biological considerations where relevant.

### 4.1 X-linked myotubular myopathy

The marked predominance of XLMTM in the reviewed literature (71.2% of patients) may reflect not only the clinical severity of this subtype, but also the substantial research interest generated by the development of disease-specific gene-replacement therapy. This therapeutic context may have encouraged dedicated natural history studies and the identification of clinical and patient-reported outcome measures to support trial readiness. Accordingly, the gene distribution observed in this review may primarily reflect the availability of relevant evidence rather than the epidemiological distribution of CM genotypes, in which RYR1-related myopathies are more frequently represented (48). The paucity of comparable neuropsychological, adaptive, and QoL data in other CM subtypes could therefore represent an important limitation to their preparedness for future clinical trials. Findings from the identified studies showed that cognitive and developmental profiles were broadly preserved in the majority of cases, consistent with the established understanding that the *MTM1* gene product, myotubularin, is primarily expressed in muscle. Nevertheless, several studies reported developmental scores at the lower limits of the normative range (24, 26) and, in a minority of cases, clinically relevant delay (29). The occurrence of structural brain anomalies in isolated cases – including Dandy-Walker malformation, progressive hydrocephalus, and cerebellar hemisphere atrophy – further suggests that CNS involvement may contribute to neuropsychological vulnerability beyond the physical burden of disease in a subset of patients. However, converging neurobiological evidence may help refine the interpretation of *MTM1* function beyond a purely muscle-restricted role. In fact, myotubularin is a ubiquitously expressed lipid phosphatase for PI(3)P and PI(3,5)P2, including in brain tissue, whose misregulation is sufficient to cause neurodegeneration in murine models (49). Consistent with this, an iPSC-based human cell model of XLMTM demonstrated that *MTM1* deficiency leads to peripheral lysosomal accumulation via *FYCO1*-mediated trafficking and *mTORC1* hyperactivation (13) – a pathway with established roles in synaptic plasticity and neurodevelopment – suggesting that the cellular mechanisms disrupted in XLMTM muscle may operate analogously in neural tissue. Notably, a recently reported XLMTM manifesting carrier with a frameshift *MTM1* mutation developed cerebral and cerebellar atrophy, cognitive impairment, brainstem dysfunction, and peripheral demyelinating neuropathy (49), extending the phenotypic spectrum of *MTM1*-related disease beyond skeletal muscle. In this context, the single case of cognitive delay reported by Abath Neto et al. (50) – excluded from this review for the absence of formal neuropsychological assessment – occurred exclusively in the patient who had sustained a pulmonary arrest at 4 months, highlighting hypoxic-ischemic injury as an alternative, potentially preventable etiology in individual cases. The finding of subclinical perseverative behavioral traits in XLMTM patients (26), though not reaching the threshold for ASD or obsessive-compulsive disorder, further suggests the possibility of a broader neuro-behavioral phenotype warranting systematic neuropsychological characterization in future prospective studies. Finally, adaptive functioning deficits documented in included XLMTM patients (VABS-II mean Adaptive IQ = 52.4) (26) were substantially below what would be predicted from intellectual functioning scores alone, strongly implicating the physical and environmental constraints imposed by severe motor and respiratory impairment as key mediators of real-world adaptive capacity. Accordingly, the moderately reduced QoL observed in these patients (PedsQL-NM mean = 50.3) (27) is consistent with this interpretation and highlights the importance of outcome measures that capture the lived experience of children with CMs beyond traditional neuropsychological metrics. Children may possess the cognitive potential for complex tasks but lack the physical or expressive means to execute them, leading to a functional deficit that hinders independence and QoL.

### 4.2 ACTA1-related congenital myopathy

Among the small number of ACTA1-related cases identified in this review, significant neuropsychological impairment was reported in two studies (32, 34). These two studies – representing all ACTA1 cases with available neuroimaging in this review – identified structural CNS abnormalities in all four patients. However, the limited number of cases and the unequal availability of neuroimaging data across genotypes preclude reliable comparisons with other CM subtypes. A possible primary neurobiological contribution is compatible with available expression data: according to the Human Protein Atlas (15), ACTA1 RNA is detectable across all major human brain regions including cerebral cortex, hippocampal formation, amygdala, cerebellum, and brainstem. This is consistent with an expression profile extending well beyond skeletal muscle. Combined with evidence that extra-muscular CNS manifestations including hypoplastic frontal lobes, lateral ventricular dilation, and corpus callosum dysgenesis are a recognized feature of ACTA1-related disease (16), these observations raise the possibility of primary CNS involvement, although secondary effects related to systemic illness, respiratory compromise, or intensive care cannot be excluded. The corpus callosum abnormalities documented across multiple ACTA1 cases included in this review (32, 34) may be compatible with a primary neurodevelopmental mechanism, given that alpha-actin is expressed across major human brain regions (15) and that brain structural anomalies in ACTA1-related disease have been demonstrated to arise prenatally, preceding any postnatal hypoxic or iatrogenic event (16).

Prenatal observations provide additional support for the possibility that CNS abnormalities may, at least in some *ACTA1*-related cases, precede postnatal hypoxic or iatrogenic events. Martínez-Diago et al. (16) described the first fetal case of nemaline myopathy due to a de novo *ACTA1* variant in which MRI at 31 weeks of gestation revealed cortical development delay and subarachnoid space enlargement, findings that definitionally precede any postnatal hypoxic or iatrogenic event. Similarly, Ueda et al. (51) reported multiple white matter foci of diffusion restriction on day four of life in a neonate with an *ACTA1* variant, without metabolic or infectious cause. Nevertheless, disentangling primary CNS involvement from hypoxic-ischemic injury remains a genuine clinical challenge: Zhao et al. (52) described periventricular signal abnormalities in a neonate with a de novo *ACTA1* variant who had also sustained severe birth asphyxia, illustrating the confounding that can obscure the genetic etiology in the acute neonatal setting. Prospective genotype-stratified cohorts and systematic prenatal neuroimaging will be essential to clarify whether, and to what extent, primary CNS involvement occurs in *ACTA1*-related disease.

### 4.3 Nemaline myopathy

Within the limited evidence available for nemaline myopathy, the largest published cohort showed a relatively favorable neuropsychological profile, with the largest published cohort (35) reporting normal intellectual functioning across 12 patients and only subtle weaknesses in auditory short-term memory. This cohort additionally described positive self-concept and well-preserved communicative abilities – an important observation given that the psychosocial sequelae of chronic pediatric neuromuscular disease are frequently underestimated in clinical practice, with adaptive functioning and mental health routinely receiving less systematic attention than motor or respiratory outcomes (53, 54). The mild language delays and borderline personal-social development observed in rarer nemaline subtypes (*TNNT1*- and *KLHL40*-related) may reflect the additive effects of physical disability, limited mobility, and reduced environmental exposure rather than primary cognitive compromise – a possibility supported by evidence that restricted independent exploration in children with physical disabilities can impair spatial and social cognition beyond what is attributable to intellectual capacity alone (17), although this cannot be resolved on the basis of the retrieved data.

### 4.4 Centronuclear myopathy

The three available centronuclear myopathy cases, each involving a different genetic background, showed heterogeneous cognitive profiles; however, they are insufficient to establish a genotype-related gradient of cognitive involvement. At one end of this spectrum, PeBenito et al. (33) reported normal intellectual functioning in a patient with centronuclear myopathy of uncharacterized molecular etiology, alongside a disharmonic profile in which verbal IQ substantially exceeded performance IQ (114 vs. 96) . A comparable disharmonic cognitive profile, albeit at a lower overall level of functioning, was observed in a case of autosomal dominant centronuclear myopathy associated with *BIN1* mutations (30), which presented with borderline global intellectual functioning (IQ = 77), reduced processing speed, and school difficulties in concentration and information processing, with preserved general language abilities. The recurrence of a disharmonic profile (VIQ > PIQ) across two genetically distinct centronuclear myopathy cases warrants cautious interpretation. A possible explanation relates to the psychometric limitations of standard IQ tests, as performance-based subtests rely heavily on visuomotor integration, manual dexterity, and processing speed (44). In this context, reduced performance scores may reflect motor and speed constraints rather than a primary deficit in non-verbal reasoning. Consistently, VIQ > PIQ discrepancies have been shown to correlate with the degree of fine motor impairment, independently of global intellectual functioning (45). Additional factors, including fatigability, reduced motor endurance, and limited opportunities for visuospatial exploration, may further contribute to this profile in children with neuromuscular disorders. In centronuclear myopathy – where proximal and distal muscle weakness, and in some cases ophthalmoplegia, may further constrain task execution – this measurement bias may represent a plausible and clinically relevant explanation. Nevertheless, a potential genotype-specific neurobiological contribution cannot be excluded. *BIN1* is expressed in neural tissue and plays a role in membrane dynamics and synaptic function (14), raising the possibility of subtle CNS involvement. However, the available evidence, limited to single-case observations, is insufficient to disentangle primary neurobiological effects from measurement artefacts, and motor-adapted neuropsychological protocols will be required to address this question.

At the more severe end of the spectrum, the SPEG-related case (37) presented with mild intellectual disability (IQ = 55) alongside mildly delayed social and language functioning. While this represents an isolated clinical observation, biological evidence raises the possibility that cognitive vulnerability in this genotype may reflect gene-specific mechanisms rather than an incidental finding: a large-scale Mendelian randomization study identified SPEG as a blood eQTL causally associated with cognitive performance at the population level (OR = 1.082, 95% CI 1.047–1.117; PPH4 = 0.950), with higher expression conferring a protective effect (55). Although this finding derives from non-myopathy cohorts and cannot be directly extrapolated to patients with loss-of-function mutations, the directionality is consistent with the observed clinical outcome. Taken together, these observations – three cases across three genetic subtypes – are insufficient to draw subtype-specific conclusions but suggest that formal neuropsychological assessment may be clinically informative in selected pediatric patients with centronuclear myopathy. The current evidence is insufficient to recommend genotype-specific surveillance protocols (18).

### 4.5 RYR1-related myopathy

The single *RYR1*-related case included in this review (23) demonstrated age-appropriate scores across all non-motor developmental domains assessed by the Ages and Stages Questionnaire at 13 months of age, with gross motor delay constituting the sole area of impairment and brain MRI within normal limits. As this evidence derives from a single infant assessed at 13 months of age, no broader conclusions regarding the neurodevelopmental profile of RYR1-related myopathy can be drawn. However, this finding may be consistent with the established biology of *RYR1*, whose encoded protein is predominantly expressed in skeletal muscle and functions principally in excitation-contraction coupling; while all three ryanodine receptor isoforms are expressed in the central nervous system, *RYR1* cerebral expression is comparatively limited – being minimal or below detection limits in hippocampal neurons in some studies – with *RYR2* representing the major neuronal isoform in the cerebral cortex and hippocampus (56).

### 4.6 SELENON-related myopathy

Among the eight pediatric patients with *SELENON*-related myopathy included in this review, formal cognitive data were available for only two individual cases (38, 39), both of whom presented with moderate intellectual disability (IQ = 45 and 48), with markedly poor academic performance reported in one case. In the six-patient pediatric cohort described by Bouman et al. (40), generic QoL was reduced across both self- and parent-proxy PedsQL ratings. Thus, while the two available cognitive profiles were among the most severe neuropsychological profiles documented across the reviewed cohort, the limited evidence precludes generalization. Nonetheless, the QoL findings point toward a consistent trend of reduced QoL within the small pediatric cohort included. This pattern may not be wholly incidental: Filipe et al. (57) demonstrated that selenoprotein N localizes to mitochondria-associated membranes; its absence causes defective ER–mitochondria contacts, impaired calcium transfer, and reduced ATP production not only in skeletal muscle but also in extramuscular cells including patient-derived fibroblasts, identifying a systemic bioenergetic deficit as a component of *SELENON*-related disease. Whether this cellular dysfunction extends to neural tissue and contributes to the observed cognitive profile remains to be established; however, disruption of mitochondria-associated membrane integrity has been implicated in the pathogenesis of several neurodegenerative disorders, including Alzheimer’s disease, Parkinson’s disease, and amyotrophic lateral sclerosis, representing a plausible mechanistic pathway warranting direct investigation in future studies (58).

### 4.7 Caregiver burden

Evidence regarding caregiver outcomes remains limited, as only three studies involving 56 caregivers were identified. Nevertheless, these studies provide preliminary indications that caregiving demands may extend beyond physical assistance. The high levels of required support documented in XLMTM caregivers (27), the variable but at times severe anxiety and depression reported by Duong et al. (28), and the substantial emotional burden emerging from the qualitative accounts of nemaline myopathy caregivers (31) suggest that caregiving may involve substantial practical, clinical, and psychological demands in some families. These observations are consistent with broader evidence from the neuromuscular literature, indicating that caregivers of children with severe congenital conditions often assume roles that extend far beyond direct physical care – encompassing monitoring of disease progression, coordination of multidisciplinary care, and acting as clinical informants for healthcare professionals – a pattern associated with increased emotional exhaustion and sustained psychological burden (59, 60). The qualitative evidence from Merchán Arjona et al. (31) provided specific illustration of this dynamic in the CM context, capturing how caregivers of children with nemaline myopathy navigate acute clinical decisions and ICU transitions with limited formal support. However, the small evidence base, heterogeneous methods, and limited representation of CM subtypes preclude broader conclusions regarding the prevalence, severity, and determinants of caregiver burden. Future studies should systematically assess caregiver mental health, QoL, support needs, and changes over time using standardized measures across genetically diverse CM populations. Emerging models of family-centered care increasingly recognize the need to address caregivers’ instrumental, self-care, and mental health needs as distinct and co-equal components of comprehensive support (61), a framework that warrants systematic adoption in the management of pediatric CMs.

### 4.8 Limitation and future directions

The current body of evidence is characterized by several major limitations. First, the overall quality of evidence is constrained by the predominance of low-level study designs, particularly case reports or case series (72.2%) and the majority classified as OCEBM Level 4 (77.8%), precluding robust causal inferences and reliable prevalence estimates. In addition, no prospective studies using standardized neuropsychological protocols and appropriate control groups were identified. Neuropsychological and psychosocial outcomes were often reported as secondary endpoints, resulting in incomplete assessment across key domains and highlighting the absence of standardized evaluation frameworks for pediatric CMs. Moreover, marked heterogeneity in instruments, age ranges, outcome definitions and reporting metrics limited comparability across studies and precluded meta-analysis. Consequently, findings were synthesized descriptively, and subgroup differences could only be explored qualitatively.

The study population was highly unbalanced, with a marked over-representation of XLMTM (71.2%) and limited data on other subtypes, often represented by single cases, restricting genotype-specific conclusions. The marked male predominance (88.5%), largely driven by the over-representation of XLMTM, further limits the generalizability of the findings and makes it difficult to disentangle potential sex-related effects from genotype-related differences.

Furthermore, longitudinal data were lacking, preventing conclusions on developmental trajectories and on the relative contribution of genetic, neurological, and disease-related factors. School outcomes were reported for only three patients, representing a major evidence gap, while caregiver trajectories were also rarely assessed. Similarly, QoL data were limited to patients with XLMTM and SELENON-related myopathy and therefore cannot be generalized to the broader CM population.

Finally, despite a comprehensive search strategy, relevant studies may have been missed; this risk may be particularly relevant for phenotype-focused studies in which neuropsychological or QoL outcomes were reported only incidentally and were not highlighted in titles, abstracts, or indexing terms.

Future research should prioritize prospective, multicenter studies with larger and genetically diverse cohorts, aiming to develop standardized neuropsychological protocols tailored to pediatric CMs. Particular emphasis should be placed on the use of motor-reduced or motor-free instruments (e.g., Raven’s Progressive Matrices, Leiter scales), language tasks minimizing speech production demands, such as picture-based receptive vocabulary, sentence comprehension, or phonological discrimination/non-word discrimination tasks, and functional measures such as adaptive behavior to more accurately capture cognitive, linguistic, and everyday abilities, especially in more severe cases. School participation, educational attainment, and QoL across genetically diverse CM subtypes should also be included as core outcomes.

Lastly, longitudinal designs will be essential to define developmental trajectories, identify early risk factors and inform clinical, rehabilitative and educational strategies. In the context of emerging disease-modifying therapies, the integration of neuropsychological, adaptive and quality-of-life outcomes into clinical trials will be critical.

## 5 Conclusion

This systematic review shows that neuropsychological and psychosocial dimensions of pediatric CMs are clinically relevant but remain substantially understudied. While many children exhibit preserved intellectual functioning, a meaningful subset presents with global developmental delay, language difficulties, adaptive impairment, neuropsychiatric features, and reduced QoL with variability across clinical conditions. Notably, QoL data were available only for patients with XLMTM and SELENON-related myopathy and cannot be generalized to other CM subtypes.

Importantly, adaptive functioning may be disproportionately affected relatively to cognitive abilities reflecting the impact of motor and language limitations, respiratory dependence and environmental constraints on everyday functioning.

Caregiver outcomes are substantial and multidimensional, underscoring the need to integrate both patient- and family-centered outcomes into multidisciplinary care

Future prospective, multicenter studies using standardized, motor-adapted neuropsychological protocols comprehensive of educational and adaptive outcomes are needed particularly in the context of emerging disease-modifying therapies.

## Data Availability

The original contributions presented in the study are included in the article/Supplementary material, further inquiries can be directed to the corresponding author.
