# Supplementary material for: Neuropsychological functioning and quality of life in congenital myopathies: a systematic review of children and caregiver outcomes
Source: Front Med (Lausanne). 2026 Aug 6;13:1876262. doi: 10.3389/fmed.2026.1876262 (PMC13490095; doi:10.3389/fmed.2026.1876262)
Supplement: Supplementary file 2 [file Table_1.docx]

***Supplementary Material 2***

# Table S1. Database-specific search strategies and applied filters

| **Database** | **Filters applied** | **Search strategy** |
| --- | --- | --- |
| *PubMed* | N/A | **Primary search:**  (("congenital myopathy"[All Fields] OR "congenital myopathies"[All Fields] OR "nemaline myopathy"[All Fields] OR "central core disease"[All Fields] OR "multiminicore disease"[All Fields] OR "centronuclear myopathy"[All Fields] OR "myotubular myopathy"[All Fields]) AND ("cognition"[All Fields] OR "cognitive"[All Fields] OR "neuropsychology"[All Fields] OR "neuropsychological"[All Fields] OR "psychological"[All Fields] OR "executive function"[All Fields] OR "attention"[All Fields] OR "memory"[All Fields] OR "learning"[All Fields] OR "language"[All Fields] OR "neurocognitive"[All Fields] OR "IQ"[All Fields] OR "intellectual"[All Fields] OR "quality of life"[All Fields] OR "QoL"[All Fields] OR "well-being"[All Fields] OR "emotional"[All Fields] OR "behavio*r"[All Fields] OR "social*"[All Fields] OR "caregiver"[All Fields] OR "communic*"[All Fields]) AND ("child"[MeSH Terms] OR "adolescent"[MeSH Terms]  OR child*[All Fields] OR pediatric*[All Fields] OR paediatric*[All Fields] OR adolescent*[All Fields]))  **Secondary search:**  ("RYR1-related" OR "ACTA1-related" OR "NEB-related" OR "MTM1-related" OR "DNM2-related" OR "BIN1-related" OR "SELENON-related" OR "SEPN1-related" OR "TPM2-related" OR "TPM3-related" OR "TNNT1-related" OR "KLHL40-related" OR "KLHL41-related" OR "LMOD3-related" OR "KBTBD13-related" OR "SPEG-related" OR "TTN-related" OR "MYH7-related" OR “X-related”) AND ("cognition" OR "cognitive" OR "neuropsychology" OR "neuropsychological" OR "psychological" OR "executive function" OR "attention" OR "memory" OR "learning" OR "language" OR "neurocognitive" OR "IQ" OR "intellectual" OR "quality of life" OR "QoL" OR "well-being" OR "emotional" OR "behaviour" OR "social*" OR "caregiver" OR "communic*") AND (child OR children OR pediatric OR paediatric OR adolescent) |
| *Scopus* | Title, abstract, key-words | **Primary search:**  TITLE-ABS-KEY ( ( "congenital myopathy" OR "congenital myopathies" OR "nemaline myopathy" OR "central core disease" OR "multiminicore disease" OR "centronuclear myopathy" OR "myotubular myopathy" ) AND ( "cognition" OR "cognitive" OR "neuropsychology" OR "neuropsychological" OR "psychological" OR "executive function" OR "attention" OR "memory" OR "learning" OR "language" OR "neurocognitive" OR "IQ" OR "intellectual" OR "quality of life" OR "QoL" OR "well-being" OR "emotional" OR "behaviour" OR "social*" OR "caregiver" OR "communic*" ) AND ( child OR children OR pediatric OR paediatric OR adolescent ) )  **Secondary search:**  TITLE-ABS-KEY ( ( "RYR1-related" OR "ACTA1-related" OR "NEB-related" OR "MTM1-related" OR "DNM2-related" OR "BIN1-related" OR "SELENON-related" OR "SEPN1-related" OR "TPM2-related" OR "TPM3-related" OR "TNNT1-related" OR "KLHL40-related" OR "KLHL41-related" OR "LMOD3-related" OR "KBTBD13-related" OR "SPEG-related" OR "TTN-related" OR "MYH7-related" OR “X-related” ) AND ( "cognition" OR "cognitive" OR "neuropsychology" OR "neuropsychological" OR "psychological" OR "executive function" OR "attention" OR "memory" OR "learning" OR "language" OR "neurocognitive" OR "IQ" OR "intellectual" OR "quality of life" OR "QoL" OR "well-being" OR "emotional" OR "behaviour" OR "social*" OR "caregiver" OR "communic*" ) AND ( child OR children OR pediatric OR paediatric OR adolescent ) ) |
| *Web of Science* | Topic | **Primary search:**  TS=((“congenital myopathy” OR “congenital myopathies” OR “nemaline myopathy” OR “central core disease” OR “multiminicore disease” OR “centronuclear myopathy” OR “myotubular myopathy”) AND (“cognition” OR “cognitive” OR “neuropsychology” OR “neuropsychological” OR “psychological” OR “executive function” OR “attention” OR “memory” OR “learning” OR “language” OR “neurocognitive” OR “IQ” OR “intellectual” OR “quality of life” OR “QoL” OR “well-being” OR “emotional” OR “behaviour” OR “social*” OR “caregiver” OR “communic*”) AND (child OR children OR pediatric OR paediatric OR adolescent))  **Secondary search:**  TS=(("RYR1-related" OR "ACTA1-related" OR "NEB-related" OR "MTM1-related" OR "DNM2-related" OR "BIN1-related" OR "SELENON-related" OR "SEPN1-related" OR "TPM2-related" OR "TPM3-related" OR "TNNT1-related" OR "KLHL40-related" OR "KLHL41-related" OR "LMOD3-related" OR "KBTBD13-related" OR "SPEG-related" OR "TTN-related" OR "MYH7-related" OR “X-related”) AND ("cognition" OR "cognitive" OR "neuropsychology" OR "neuropsychological" OR "psychological" OR "executive function" OR "attention" OR "memory" OR "learning" OR "language" OR "neurocognitive" OR "IQ" OR "intellectual" OR "quality of life" OR "QoL" OR "well-being" OR "emotional" OR "behaviour" OR "social*" OR "caregiver" OR "communic*") AND (child OR children OR pediatric OR paediatric OR adolescent)) |
