# Supplementary material for: Neuropsychological functioning and quality of life in congenital myopathies: a systematic review of children and caregiver outcomes
Source: Front Med (Lausanne). 2026 Aug 6;13:1876262. doi: 10.3389/fmed.2026.1876262 (PMC13490095; doi:10.3389/fmed.2026.1876262)
Supplement: Supplementary file 3 [file Table_2.docx]

***Supplementary Material 2***

# Table S2. Genetic variants identified in patients with congenital myopathies included in the review

| **Study**  **(First author, year)** | **Subjects (N=)** | **Gene** | **Variant** | **Protein Notation** | **Zygosity** | **Consequence of mutation** | **Notes** |
| --- | --- | --- | --- | --- | --- | --- | --- |
| *Alkhunaizi et al., 2019* | 1 | RYR1 | c.14545G>A + c.14598G>C | p.Val4849Ile + p.Met4866Ile | Compound heterozygous | Missense | Paternal + maternal; Sanger confirmed |
| *Barreto-Mota et al., 2023* | 1 | MTM1 | c.722G>A | p.Arg241His | Hemizygous | Missense | Likely pathogenic (ACMG); maternal inherited |
| *Bouman et al., 2023* | 6 | SELENON | c.713dup + c.1332_1334del  c.943G>A  c.1A>G  c.943G>A + c.301G>C  c.713dup + c.13_22dup  c.943G>A | p.Asn238fs + p.Asn444del  p.Gly315Ser  p.Met1?  p.Gly315Ser + r.(spl?)  p.Asn238fs + p.Gln8fs  p.Gly315Ser | Compound heterozygous  Homozygous  Homozygous  Compound heterozygous  Compound heterozygous  Homozygous | Frameshift + in-frame del.  Missense  Loss-of-function (start codon)  Missense + splice-site  Frameshift + frameshift  Missense | — |
| *Buchignani et al., 2024* | 1 | KLHL40 | c.1582G>A | p.Glu528Lys | Homozygous | Missense | — |
| *Cumbo et al., 2024* | 9 | MTM1 | NR | NR | NR | NR | Variants not individually specified |
| *Dowling et al., 2022* | 34 | MTM1 | NR | NR | NR | Nonsense 50%; missense 35.3%; in-frame deletion 14.7% | — |
| *Herman et al., 1999* | 30 | NR | NR | NR | NR | Frameshift 33.3%; missense 23.3%; deletion 16.7%; splicing 6.7%; nonsense 6.7%; complex 13.3% | — |
| *Kouwenberg et al., 2017* | 1 | BIN1 | c.53T>A | p.Val18Glu | Heterozygous | Missense | — |
| *Mohamadian et al., 2020* | 1 | SELENON | c.1446delC | p.Asn483Thrfs*11 | Homozygous | Frameshift | Consanguineous family; WES + Sanger confirmed |
| *Nóbrega et al., 2024* | 1 | ACTA1 | c.169G>C | p.Gly57Arg | NR | Missense | — |
| *PeBenito et al., 1978* | 1 | NR | NR | NR | NR | NR | Genetic data not reported |
| *Saito et al., 2011* | 3 | ACTA1 | c.801G>C; c.283C>A; c.430C>T | p.Gly270Arg; p.Asn94Lys; p.Leu144Phe | De novo (dominant) | Missense | Three independent patients |
| *Wallgren-Pettersson et al., 1989* | 12 | NR | NR | NR | NR | NR | Genetic data not reported |
| *Wang et al., 2023* | 1 | TNNT1 | c.587dupA + c.387+5G>A | p.Asp196Glufs*41 + exon 9 skipping | Compound heterozygous | Frameshift + splicing | Sanger confirmed |
| *Yildirim et al., 2021* | 1 | SPEG | c.153C>T | p.Asn51= | Homozygous | Synonymous (splice-affecting) | Novel; WES + Sanger confirmed |
| *Zhang et al., 2021* | 1 | SELENON | c.1574T>G + c.1286_1288delCCT | p.Ser429del | Compound heterozygous | In-frame deletion | Novel variant |

***Note.*** Detailed overview of genetic findings across included studies, reporting gene, cDNA and protein variants, zygosity, and variant type for each patient or cohort. When available, additional information on variant classification and inheritance is provided. NR: not reported. Variants were described according to the original studies.
